# Détermination des paramètres bioécologiques et entomologiques d’*Anopheles gambiae sl* dans la transmission du paludisme à Bandundu-ville, République Démocratique de Congo

**DOI:** 10.11604/pamj.2015.22.108.6774

**Published:** 2015-10-08

**Authors:** Emery Metelo Matubi, Eric Bukaka, Trésor Bakambana Luemba, Hyppolite Situakibanza, Ibrahim Sangaré, Gauthier Mesia, Dieudonné Mumba Ngoyi, Nguya Kalemba Maniania, Charles Ngandote Akikwa, Jean Pierre Basilua Kanza, Jean-Jacques Muyembe Tamfum, Jonas Nagahuedi Bongo sudi

**Affiliations:** 1 Institut National de Recherche Biomédicale (INRB/Kinshasa), B.P 1197 KIN 1, Kinshasa, République Démocratique de Congo; 2 Faculté de Médecine, Université de Bandundu (UNIBAND), B.P 548 Bandundu-ville, Bandundu, République Démocratique de Congo; 3 Faculté des Sciences, Département de Biologie, Unité de Recherche Entomologique, B.P 190 KIN XI, Université de Kinshasa (UNIKIN), République Démocratique de Congo; 4 Faculté de Médecine, B.P 834 KIN XI, Université de Kinshasa (UNIKIN), République Démocratique de Congo; 5 Service de Parasitologie-mycologie, INSSA de Bobo-Dioulasso, Burkina Faso; 6 International Centre of Insect Physiology and Ecology (ICIPE), P.O. Box 30772 - 00100 GPO, Nairobi, Kenya; 7 Croix-Rouge Françoise en RD Congo(CRf-RDC), Kinshasa et Institut Supérieur des Techniques Médicales de Kenge (ISTM/Kenge) B.P 8631 KIN, Bandundu, République Démocratique de Congo; 8 Institut Supérieur des Techniques Médicales de Kinshasa (ISTM/Kinshasa), B.P 774 KIN XI, RD Congo

**Keywords:** Paramètres entomologiques, bioécologiques, anopheles gambiae, entomological parameters, bioecology, anopheles gambiae

## Abstract

**Introduction:**

La présente étude a été menée à Bandundu-ville (RDC) en vue d'identifier les paramètres écologiques et entomologiques modulant la transmission du paludisme ainsi que leur tendance saisonnière dans cette agglomération.

**Méthodes:**

Cette étude a été réalisée dans la période du 1^er^ juin au 31 décembre 2011. Des prospections des gîtes larvaires d'anophèles avec récolte ont été réalisées, les paramètres physiques, physico-chimiques et environnementaux déterminés. La densité larvaire a été estimée selon une échelle de classes de densité, inspirée de la méthode de Carron pour chaque type de gîtes. Quarante-huit maisons ont été sélectionnées et prospectées pour la récolte des moustiques par pulvérisation intradomicilaire. L'identification des moustiques a été faite sur base des critères morphologiques de Gilles et Demeillon. L'Indice sporozoïtique (Is) a été déterminé par le test ELISA CSP de *Plasmodium falciparum* à l'Institut National de Recherche Biomédicale selon le protocole de Robert Wirtz. Les autres paramètres entomologiques comme la densité, le taux d'agressivité, le taux d'inoculation entomologique (TIE) ainsi que l'indice de stabilité ont été déterminés selon le protocole de l'OMS. La régression linéaire a été réalisée au seuil de signification de 0,05 pour identifier les déterminants de la densité larvaire.

**Résultats:**

Cent-sept gîtes larvaires ont été identifiés et caractérisés en 5 types (digues et puits d'eau, collections d'eau maraîchère et concasseurs moellons, marais Régie de distribution d'eau, marais le long des rivières et ruisseaux et flaques d'eau de pluies). La densité larvaire moyenne a été de 117,4±64,1. Quatre mille cinq cents quatre-vingt-huit moustiques ont été capturés et identifiés, parmi lesquels 1.258 *Anopheles gambiae sl* avec une densité de 8,86, un taux d'agressivité de 1,55 piqûre par homme par nuit, l'Is de 5,6%, un TIE de 0,085 piqûre infectante par homme par nuit, l'espérance de vie moyenne d'anophèles de 16,4 jours et un indice stabilité de 6,512. L'analyse des données a montré que la superficie des gîtes larvaires influençait significativement la densité larvaire (p < 0,001). Par contre, la turbidité et la conductivité des gîtes influençaient négativement la densité larvaire (p < 0,05, IC 95%).

**Conclusion:**

Les diverses biotopes, la forte densité d’*Anopheles gambiae sl*, le TIE et l'indice de stabilité placent Bandundu-ville en zone endémique stable.

## Introduction

L'anophèle est l'arthropode le plus dangereux du monde, vecteur du paludisme qui continue d’être la principale cause de morbidité et de mortalité en Afrique subsaharienne [1]. Chaque année 207 millions de personnes souffrent du paludisme dans le monde [1]. Près de 627.000 enfants et adultes meurent de cette maladie chaque année malgré l'existence de mesures préventives et curatives efficaces utilisés dans les zones endémiques [1–3]. Sur plus de 3000 espèces d'anophèles enregistrées dans le monde, seuls environ 60 transmettent le paludisme. En Afrique, les principaux vecteurs sont *An. gambiae, An. funestus, An. nili et An. Moucheti*. Ces anophèles appartiennent tous à des complexes d'espèces [4]. Ces anophèles peuvent avoir des capacités vectorielles et des comportements très variables. La République Démocratique du Congo (RDC), comme l'ensemble des pays d'Afrique noire, paie un lourd tribut au paludisme. Sa situation géographique particulière et la diversité de ses climats en font toute son originalité. Etiré sur une superficie d'environ 2.345.000 km^2^, ce pays présente en effet la quasi-totalité des strates bioclimatiques rencontrées en Afrique, des savanes sahéliennes aux forêts équatoriales [5, 6]. Cependant, cette hétérogénéité ne permet pas d'appréhender le paludisme en RDC dans sa globalité. La connaissance des différents espaces Congolais (territoires, district, etc.) et la description de l’épidémiologie locale du paludisme dans ces différents milieux sont des conditions initiales à la bonne compréhension de l'endémie palustre dans ce pays. Par ailleurs le paludisme reste préoccupant en RDC notamment dans la région de Bandundu, en dépits des multiples interventions mises en marche avec l'appui du fonds mondial [5, 7]. La compréhension de cette complexité est d'une importance majeure dans la mise en œuvre de la stratégie efficace de lutte contre le paludisme tant par la lutte antivectorielle que dans tout programme d'intervention de santé publique, tels que dans l'usage de tout programme d'intervention de santé publique, par exemple dans l'usage des médicaments ou dans les essais de vaccins [4, 8, 9]. La lutte antivectorielle se fonde sur la meilleure connaissance du vecteur et l'utilisation de techniques efficaces contre les souches et dynamiques locales. Par conséquent, les efforts doivent être consentis d'abord sur la bio-écologie des vecteurs pour mieux orienter les opérations de lutte [10–13]. Cependant le manque des données sur les études entomologiques dans cette agglomération de Bandundu, constitue un handicap dans la maîtrise et l’élaboration des stratégies de contrôle efficace. La présente étude, contribue à la connaissance de l’épidémiologie locale de la transmission du paludisme et à l’élaboration de la cartographie, en vue d'améliorer la surveillance de la maladie.

## Méthodes

### Milieu d’étude

#### Situation géo-démographique

**Figure 1:**
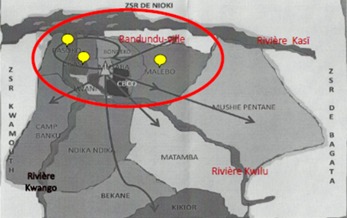
Carte de la zone de santé urbano-rurale de Bandundu

**Figure 2:**
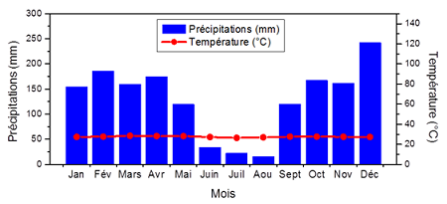
Courbe ombro thermique de Bandundu-ville 2009 -2013

Bandundu-ville est le Chef-lieu de la province de Bandundu en RDC. Elle est située entre 17°22'43"de longitude Est, 3°21'05" de latitude Sud et à 324 m d'altitude, à l'Ouest de la dite Province [14, 15]. C'est une Zone de Santé urbano-rurale, délimitée au Nord par la Zone de Santé de NIOKI, au Sud par la Zone de Santé de Kutu, à l'Est par la Zone de Santé de Bagata et à l'Ouest par la Zone de Santé de Kwamouth. Elle est à 432 Km de distance de Kikwit, à 200 Km de Kenge, les autres principales villes de cette province et à 400 Km de Kinshasa capitale de la RDC [14] (Figure 1). Bandundu-ville est en générale marécageuse baignée et entourée par les rivières Kwilu et Kwango et le confluent de la rivière Kasaï avec plusieurs ruisseaux et possède des sols limono-argileux et hydromorphes qui favorisent la création de plusieurs gîtes. Elle a une superficie de 291 Km2 avec environ 285.411 habitants et une forte densité démographique de 980,79 habitants/Km2. Administrativement, la ville est divisée en trois Communes (Basoko, Disasi, et Mayoyo) et 16 quartiers [15]. Bandundu-ville se trouve dans le climat de basse altitude, caractérisé par un climat tropical humide avec deux saisons bien marquées. La saison des pluies caractérisée par des folles chutes des pluies et une chaleur constante toute l'année et saison sèche de 4 mois ou la pluviométrie chute jusqu’à s'annuler comme le montre la courbe ombro-thermique, Figure 2 [16, 17]. La température moyenne annuelle est de 26,9°C, la pluviométrie annuelle est de 800 à 1500 mm, l'humidité moyenne annuelle est de 77% et la durée d'insolation moyenne annuelle est de 4,35 heures [16, 17].

### Matériel et méthodes

Le matériel biologique de notre étude a été constitué des anophèles capturés dans les trois Communes de Bandundu-ville. Dans chaque quartier les prospections des gîtes larvaires ont été réalisées de juin à décembre 2011, en suivant les différents itinéraires tracés pour identifier et caractériser les principaux gîtes larvaires. Cette période incluait tant la saison des pluies que la saison sèche. Des paramètres physiques, physico-chimiques (pH, conductivité, turbidité et température) ont été relevés par la sonde multi paramétrique (GOMBO Hanna HI 98129). La profondeur et surface ont été mesurées par le mètre ruban. Les larves ont été récoltées dans leur gîte au moyen d'une louche (500 ml; modèle OMS) selon la méthode de dipping [18]. Le protocole d’échantillonnage larvaire a consisté à réaliser une dizaine de plongées de louche à plusieurs niveaux du point de récolte. Après chaque coup de louche, le contenu a été transvasé dans une cuvette et le nombre de larves ont été estimées selon une échelle de classes de densité, inspirée de la méthode de Carron, variant de 1 à 5: 1, < 1; 2, 1-10; 3, 11-50; 4, 51-100; 5,> 100 [19]. Ainsi que les paramètres environnementaux (couvert végétal) ont été prélevés pour déterminer si la surface du gîte est occupée par une végétation d'une hauteur ≥ 20 cm (ombragé) et non occupée (ensoleillé). Ces prospections ont consisté à parcourir les différents itinéraires chaque semaine pendant 6 mois en vue de vérifier l'assèchement des gîtes et de caractériser les gîtes temporaires à permanents [20]. Des maisons ont été sélectionnées au hasard à partir de ces gîtes et en évitant des recoupes entre quartiers. Trois maisons ont été choisies au hasard par quartier et les personnes ayant passé nuit dans chaque maison ont été identifiées. La capture de la faune Culicidiènne résiduelle par pulvérisation aux pyréthrines a été réalisée de 6 heures à 10 heures du matin. Les draps blancs ont été étalés sur le pavement dans toutes les pièces de la maison. La pulvérisation a commencé d'abord à l'extérieur de la maison, devant les portes et fenêtres, ensuite à l'intérieur de la maison. Après 15 minutes, les portes et les fenêtres ont été ouverts pour récupérer les moustiques tombés sur les draps [21, 22]. Sur terrain, les échantillons ont été placés dans des tubes individuels numérotés et contenant le silicagel. Ils ont été acheminés à l'Institut National de Recherche Biomédicale de Kinshasa et conservés à -20 °C. Le genre et l'espèce des moustiques capturés ont été identifiés sur base de la clé dichotomique de Gilles et De meillon au microscope entomologique [23–25]. Le test ELISA circumsporozoïtique de *Plasmodium falciparum* a été réalisé sur les broyats du complexe tête-thorax des femelles d'Anopheles pour déterminer l'indice sporozoïtique, qui estime le rapport du nombre de femelles avec présence de la protéine circumsporozoïte dans le broyat tête-thorax sur le nombre de femelles testées selon le protocole de Wurtz [26]. Et les abdomens ont été disséqués sous stéréo-microscope pour déterminer l'indice de parturité sur base de trachéoles. Cet indice est obtenu par le pourcentage de femelles pares et il a été aussi possible d’établir la probabilité quotidienne de survie de la population d'anophèles sur base de cette parturité (EV = 1/-Ln P). Les paramètres entomologiques de transmission ont été estimés sur base de la méthode indirecte de l'OMS [21, 22]. Les calculs mathématiques sont basés sur les résultats de deux captures combinées. Plusieurs paramètres ont été déterminés: le taux d'inoculation (TIE) = ma. Is (où ma est le nombre des piqûres par homme et par nuit, Is est l'indice sporozoïtique) et l'indice de stabilité en se basant notamment sur les formules de Macdonald: a/-Ln p est un indice essentiellement entomologique où (a) est le nombre de repas par moustique et par homme, soit le produit de l'inverse de la durée du cycle gonotrophique par l'indice d'anthropophilie.

### Analyse des données

Les données ont été saisies et analysées à l'aide du logiciel Epi info version 3.5.1, 2008 et SPSS version 22. Les paramètres entomologiques étudiés ont été la densité anophélienne (d), le taux d'agressivité (ma), l'indice sporozoïtique (Is) et le taux d'inoculation entomologique (TIE). Le test de Chi-carré a permis une comparaison de ces paramètres entomologiques, à l'exception de la densité anophélienne entre les deux saisons (saison sèche et des pluies) et entre les sites de capture. Tandis que l’évaluation de l'Analyse de Variance a permis de comparer les paramètres entomologiques entre les sites de capture. La variation saisonnière de ces variables quantitatives a été appréciée par le test de Student. L'influence des facteurs bioécologiques sur la densité larvaire a été appréciée par la régression linaire après analyse uni variée et multi variée. La variable dépendante (densité larvaire), ainsi que les variables indépendantes (pH, T°, turbidité, conductibilité, surface et profondeur) à IC 95% au seuil de signification (0,01< p < 0,05).

## Résultats

### Les gites larvaires

Un total de 107 gîtes, caractérisés en 5 types de collection d'eau ont été identifiés (Tableau 1). Parmi le 5 types des gîtes caractérisés, les marais REGIDESO ont constitué 40,02% (n = 43/107) des gîtes et la Commune de Disasi a renfermé les plus grand nombre de gîtes (52,3% n = 56/107), confère tableau 1. Les paramètres physiques, physico-chimiques et environnementaux par gîtes d'anophèles et par commune sont présentés dans les Tableau 2,Tableau 3 (Caractéristiques physiques, physico-chimique des gîtes d'anophèles selon leur types et Couverture végétale et nature des gîtes par saison). La plupart des eaux de tous les gîtes prospectés sont neutres. Le pH basique a été enregistré dans des marais REGIDESO, qui présente des différences très hautement significatives comparativement à d'autres gîtes (p< 0,001). Il a également été observé une différence très hautement significative entre les profondeurs de différents types de gîtes (p < 0,001). Les profondeurs les plus importantes ont été enregistrées dans les Marais le long des rivières et ruisseaux. Ces derniers présentent également de fortes densités larvaires en comparaison des autres types de gîtes (p < 0,05). Cependant, la température, la conductivité et la turbidité ne présentent pas de différence significative quant aux différents gîtes (p> 0,05), confèreTableau 2. Quant à la température, elle est plus élevée à Mayoyo et présente en effet une différence significative comparativement à deux autres communes (F_(2,104)_ = 3,512, p = 0,033). Mayoyo se caractérise également par de faibles valeurs de conductivité (F_(2,104)_ = 4,736, p = 0,011) et de turbidité (F_(2,104)_ = 5,816, p = 0,004) par rapport à Basoko et Disasi. La plupart des gîtes larvaires d’*Anopheles gambiae sl* à Bandundu-ville sont ensoleillés et permanents. Et la saison des pluies est donc la meilleure pourvoyeuse des gîtes larvaires (Tableau 3).

**Tableau 1:** Types de gîtes par commune

Type de gîtes	COMMUNE			
	BASOKO	DISASI	MAYOYO Total (n = 107)	
Digues et puits d'eau	6(5.6%)	4(3.7%)	1(0.9%)	11(10.3%)
Collections d'eau maraîcher et concasseurs moellons	1(0.9%)	14(13.1%)	2(1.9%)	17(15.9%)
Marais REGIDESO	16(15%)	22(19.6%)	5(4.7%)	43(40.2%)
Marais le long des rivières et ruisseaux	8(7.5%)	7(6.5%)	7(6.5%)	22(20.6%)
Flaque d'eau des pluies	0(0%)	9(8.4%)	5(4.7%)	14(13.1%)
TOTAL	**31(29%)**	**56(52.3%)**	**20(18.7%)**	**107(100%)**

**Tableau 2:** Caractéristiques physiques et physico-chimiques des gîtes d'anophèles

Type de gîtes	pH	Température (°C)	Conductivité (µs/cm)	Turbidité (ppm)	Profondeur (cm)	Superficie (m^2^)	Densité larvaire
1. Digues et puits d'eau	7,6 ± 0,4	27,6 ± 1,2	439,5 ± 94,8	252,7 ± 93,6	22± 3,29	1,4 ± 0,5	101,1 ± 41,3
2. Collections d'eau maraîchère et concasseurs moellons	7,5 ± 0,6	26,3 ± 2,7	448, 7 ± 112,9	267,8 ± 80,1	24,35 ± 4,54	1,4 ± 0,5	90,1 ± 61,2
3. Marais REGIDESO	8,1 ± 0,5	28,0 ± 2,3	395,6 ± 124,9	209,1 ± 67,2	10,43 ± 1,24	1,2 ± 0,6	120,6 ± 62,8
4. Marais long des rivières et ruisseaux	7,4 ± 0,6	27,3 ± 2,1	426,9 ± 290,1	229,6 ± 77,3	63,68 ± 14,32	1,6 ± 0,7	165,8 ± 109,7
5. Flaque d'eau des pluies	7,4 ± 0,5	27,8 ± 2,6	389,6 ± 186,7	232,8 ± 101,1	15,43 ± 1,87	1,8 ± 0,6	109,6 ± 45,4
F_(4,102)_	10,548***	1,805 (ns)	0,437 (ns)	1,946 (ns)	227,89***	3,417*	3,245*
p-value (α = 0,05)	3,426E-7	0,134	0,782	0,109	6,467E-5	0,012	0,015

Légende: p > 0,05, différence non significative (ns)

* 0,01 < p<0,05, différence significative

0,001 < p<0,01, différence hautement significative (**)

*** p < 0,001, différence très hautement significative

**Tableau 3:** Couverture végétale et nature des gîtes par saison

Types des gîtes	Couverture végétale		Nature des gîtes		Saison (n = 107)	
	Ensoleillé	Ombragé	Permanent	Temporaire	Sèche	Pluvieuse
Digues et puits d'eau	8(7, 5%)	3(2, 8%)	5(4, 7%)	6(5, 6%)	6(5, 6%)	5(4, 7%)
Collections d'eau maraîchère et concasseurs moellons	15(14%)	2(1, 9%)	13(12, 1%)	4(3, 7%)	10(9, 3%)	7(6, 5%)
Marais REGIDESO	29(27, 1%)	14(13, 1%)	22(20, 6%)	19(17, 8%)	13(12,1%)	30(28, 0%)
Marais long des rivières et ruisseaux	17(15, 9%)	5(4, 7%)	15(14%)	7(6, 5%)	12(11,2%)	10(9, 3%)
Flaque d'eau de pluies	9(8, 4%)	5(4, 7%)	0(0%)	14(13, 1%)	0(0,0%)	14(13, 1%)
**Total**	**78(72, 9%)**	**29(27, 1%)**	**57(53, 3%)**	**50(46, 7%)**	**41(38,3%)**	**66(61, 7%)**

### Paramètres entomologiques

#### Capture

**Figure 3:**
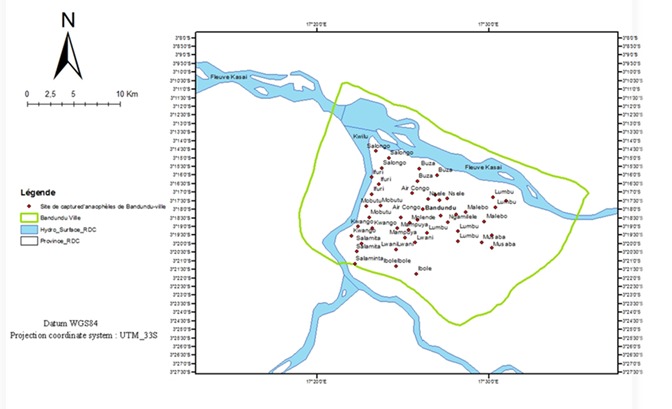
Carte des points de capture d'Anophèles à Bandundu-ville

**Figure 4:**
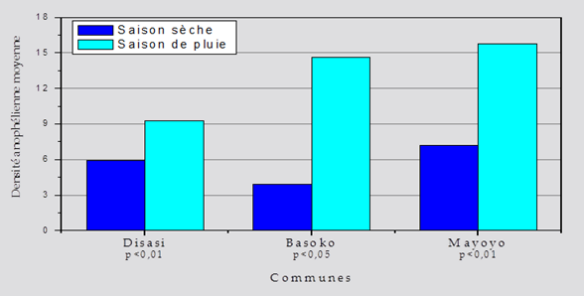
Tendance saisonnière de la densité anophèlienne par commune

Les coordonnées géographiques de chaque point de capture (48 maisons pour capture) ont été relevées à l'aide d'un GPS de la marque Garmin G Maps 72. Ainsi que l'adresse de l'habitation (quartier, rue et numéro) a été intégrée à une base de données SIG pour élaborer la carte (Figure 3). Deux périodes de capture ont été réalisées pour un total de 4.588 moustiques capturés par pulvérisation aux pyréthrines dans les 3 communes de Bandundu-ville. Il a été répartis en 1.258 anophèles et 3.330 culex ont été identifiés (Tableau 4). Il ressort de ce tableau que la commune de Disasi est celle qui a présenté un nombre élevé d'anophèles (44% n = 562/1279), la commune de Basoko a présenté un nombre élevé d'Anopheles gambiae sl gorgé (25,6% n = 187/729). Et la commune de Disasi est la plus étendu, ayant enregistré plus de quartiers (50% n = 8/16), de maisons (50% n = 24/48) et plus de personnes (45,5% n = 107/235) qui ont effectivement passé nuit au moment de capture (Tableau 4). Il existe une différence très significative entre les effectifs d'anophèles capturés pendant la saison sèche et la saison des pluies (X^2^; 16,268 < 173,429; p = 0,001). La densité anopheliènne varie d'une commune à une autre mais cette différence n'est pas statistiquement significative tant à la saison sèche (F_(2,13)_=0,491; p = 0,623) qu’à la saison des pluies (F_(2,13)_=0,954 p = 0,411). Cependant, la densité anopheliènne moyenne par saison aurait connu une influence saisonnière (5,69 à la saison sèche et 13,22 à la saison des pluies, t = 5,112 p < 0,001) (Figure 4).

**Tableau 4:** *Anopheles gambiae sl* capturé par saison

Commune	Maison	Personne	Saison							
			Sèche				Pluies			
			Femelle	Mâle	Gorgé	Non gorgé	Femelle	Mâle	Gorgé	Non Gorgé
DISASI	24	107	142	99	118	24	223	98	181	42
BASOKO	15	90	59	21	46	13	219	125	203	16
MAYOYO	9	38	65	14	60	5	142	72	127	15
**Total**	**48**	**235**	**266**	**134**	**224**	**42**	**584**	**295**	**505**	**73**

##### Taux d'agressivité

L'agressivité anophélienne présente à la saison sèche des moyennes comparables entre les communes (X^2^=4,433, p = 0,109). Elle a été significativement différente à la saison des pluies (X^2^ = 9,913; p = 0,007). Cependant, le taux d'agressivité moyen de Bandundu-ville connaît l'influence de la variation saisonnière (X^2^= 0,501, p = 0,776) (Tableau 5).

**Tableau 5:** Paramètres entomologiques par saison

Commune	Saison							
	Sèche				Pluies			
	Densité	ma	Is	TIE	Densité	ma	Is	TIE
Disasi	5,92	1,1	11	0,12	9,29	1,69	3	0,05
Basoko	3,92	0,51	4	0,02	14,60	2,26	5	0,11
Mayoyo	7,22	1,58	14	0,22	15,78	3,34	4	0,13
Moyenne	**5,69**	**1,06**	**9,67**	**0,12**	**13,22**	**2,43**	**4**	**0,10**

### Indice sporozoïtique

**Figure 5:**
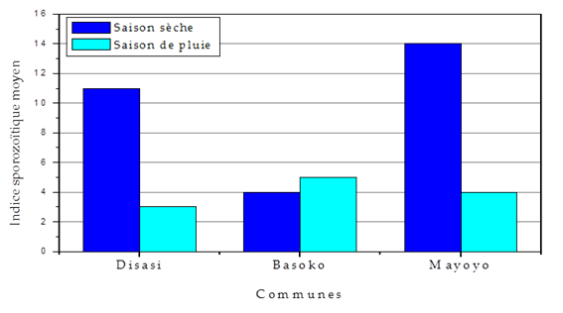
Tendance saisonnière de l'indice sporozoïtique par commune

Il a été observé une différence significative entre l'indice sporozoïtique des communes tant à la saison sèche qu’à la saison des pluies (p < 0,001), sauf à Basoko, ou il n'existe pas de différence significative. Il n'existe pas de différence significative entre l'Is de la saison sèche et des pluies, dont l'indice sporozoïtique moyen de Bandundu-ville ne subit pas l'influence saisonnière (X^2^= 3,852, p = 0,146), voir Figure 5.

### Taux d'inoculation entomologique

Le taux d'inoculation entomologique a été légèrement élevé en saison sèche à Disasi et Mayoyo, suite au phénomène de lessivage par proximité aux cours d'eau tandis qu'une situation inverse est observée à Basoko (Tableau 5).

### Indice de stabilité

Le cycle gonotrophique considéré dans notre étude a été égal à 2,5 jours. Quant à l'anthropophilie, nous avons travaillé sur la faune résiduelle qui a révélé que les moustiques piquent l'homme à l'intérieur des habitations. Cet indice peut être considéré égal à 99%. La parturité a été de 0,858, a = 0,396, p = 0,941, l'espérance de vie moyenne d'anophèles (EV) est 16,4 jours et l'indice de stabilité est de 6,512.

### Détermination des facteurs bioécologiques influençant la densité larvaire anophélienne

La régression linéaire a permis d'identifier les déterminants de la densité larvaire anophélienne. Le coefficient de détermination R^2^ compare les valeurs estimées de la variable dépendante (densité larvaire anophélienne) à ses valeurs observées (Tableau 6). L'analyse uni variée des paramètres de turbidité, conductivité et superficie ont influencé significativement la densité larvaire au seuil (0,01< p < 0,05). Mais cette influence a été dans le sens négatif dont la turbidité, la conductivité et la superficie faibles ont un effet significatif sur l'augmentation de la production larvaire. La température et la profondeur bien que soient non significatives au seuil (p > 0,05), mais influencent la densité larvaire. La température a une influence positive sur la densité larvaire et la profondeur influence négativement. Après l'analyse multi variée seule la superficie a effet significatif sur la densité larvaire au seuil (p < 0,001), voir Tableau 6.

**Tableau 6:** Les facteurs bioécologiques déterminant la densité larvaire

Caractéristiques	Analyse uni variée		Analyse multi variée		
	Coéffient R.	P valeur	Coéfficient R.	95% IC	P valeur
Température (°C)	2,26	0,47	3,35	-2,91 à 9,61	0,29
Ph	4,17	0,74	-13,39	-38,92 à 12,15	0,30
Turbidité (ppm)	-0,19	0,03*	-0,06	-0,30 à 9,61	0,62
Conductivité (µs/cm)	-0,10	0,02*	-0,06	-0,17 à 0,05	0,28
Superficie (m^2^)	-46,03	<0,001*	-43,41	-64,85 à-21,96	<0,001*
Profondeur (cm)	-0,46	0,1	-0,31	-0,94 à 0,32	0,34

* Légende: 0,01 < p<0,05, différence significative

## Discussion

D'après Mouchet et *al*, les différentes espèces d'anophèles exploitent une grande variété de collections d'eau comme gîtes, notamment les mares résiduelles des surfaces stagnantes ensoleillées, mares à végétation dressée, eaux saumâtres etc. L’*An. gambiae sl* utilise de façon préférentielle des mares résiduelles des surfaces stagnantes ensoleillées dans plusieurs régions d'Afrique [27–30]. La majorité des gîtes larvaires (72,9% n = 78/107) de Bandundu-Ville ont été des mares résiduelles ensoleillées et ont été exploités par l’*An.gambiae sl*. Les gîtes larvaires d'anophèles sont nombreux et éparpillés dans les trois communes de Bandundu-ville. Et la majorité de ces gîtes sont créés suite aux activités anthropiques. La présence des *An. gambiae sl* à Bandundu-ville en saison sèche a été clairement associée à la productivité et à la permanence des gîtes larvaires, qui sont liées aux activités anthropiques et l'urbanisation. Cette situation illustre les exceptionnelles capacités de ces An. gambiae sl à exploiter ces ressources, en utilisant les aménagements hydro-agricoles [31, 32]. C'est le cas notamment au Sénégal dans la zone urbaine de Dakar où existent plusieurs milliers de puits [30] à l'ouest du Kenya où la majorité des gîtes ont été créés par les activités de l'homme [33]. Dans la présente étude 40,2% des gîtes ont été créés par la dégradation du réseau d'eau potable (REGIDESO/Bandundu) mal entretenu. Une telle situation a été observée à Kinshasa par Karch [31]. Un phénomène d'adaptation ou de réinstallation du vecteur dans la ville exploitant les diverses petites mares créées par les fuites. L'importance d’*An. gambiae sl* par rapport aux autres anophèles dans la zone de Bandundu-ville est clairement établie. L’*An. gambiae sl* a été l'unique espèce observée et responsable de la transmission du paludisme à Bandundu-ville. La même situation a été observée à Kinshasa [31] et dans d'autres villes de RDC (Kingasani, Bolenge, Kimpese et Katana) [34]. Au Cameroun: à Nditam et à Ngoumé [32], au Madagascar [23] et dans deux village de l'Ouest de Kenya (Ternan et Lunyerere) [33]. De même selon le rapport de l'OMS [2] et la cartographie mondiale des anophèles vecteurs du paludisme [35]. Ils contrastent avec la présence majoritaire d’*An. gambiae sl*. et présence secondaire de l’*An. funestus, An. moucheti et An. Nili* comme vecteur du paludisme en R.D. Congo, suite à une différence écologique déterminant le biotope larvaire [6, 7]. La conductivité et la turbidité faibles des gîtes ont une influence sur la densité larvaire, ainsi que les paramètres entomologiques de la transmission du paludisme. Des observations similaires ont été faites à Banambani au Mali où La conductivité et le TDS ont eu des effets significatifs sur la division des gîtes et production des larves [29, 36] [37].

La conductivité et la turbidité des gîtes larvaires ont été élevées à Bandundu-ville et de ce fait ont une influence négative sur la densité. La transmission du paludisme est pérenne à Bandundu-ville à cause de la permanence des gîtes aussi bien pendant la saison des pluies que pendant la saison sèche et de la présence de l’*An. gambiae sl*, ainsi que son inféctivité aux plasmodies [38–40]. La densité anophélienne endophile est élevée pendant la saison de pluies grâce à la production larvaire et à la multiplication des collections d'eau qui se transforment en gîtes (61,7% n = 66/107). Cette situation est décrite dans la majorité d’études en zone tropicale [29, 31, 41, 42]. L'indice sporozoïtique a été plus élevé pendant la saison sèche (9%) que pendant la saison de pluies (4,1%), malgré la forte densité anophélienne observée pendant la saison des pluies. Cela pourrait se justifier par le fait que la densité des vecteurs a tendance à baisser suite aux interventions de lutte ou au changement climatique et la capacité vectorielle individuelle des survivants peut être plus grande [29, 36]. L'indice sporozoïtique moyen de 5,6% a été plus élevé à Bandundu-ville par apport à celui trouvé à Kinshasa (3,3%) [6, 31]. Sans doute pour des raisons de différence écologique. Le taux d'agressivité moyen (1,55 piqûre/homme/nuit) et le taux d'inoculation entomologique moyen (0,085 piqûre infectante/homme/nuit) ont été inférieurs à ceux trouvés à Kinshasa, 16,28 piqûres/homme/nuit et 0,54 piqûre infectante/homme/nuit respectivement [31]. L'Esperance de vie moyenne d'anophèles trouvée a été très supérieur (16,4 jours) par apport à celle trouvée par Karch à Kinshasa (8,5 jours) et Mulumba à Kinshasa (12,8 jours), cette différence est surement due aux conditions climatiques et écologiques très favorables au développement d'anophèles à Bandundu-ville [31, 39]. L'indice de stabilité de 6,512 a Bandundu-ville a été plus élevé par apport celui observé à Brazzaville (5,04) en République du Congo [43, 44] et Kinshasa (3,50) [31]. Le paludisme est une maladie loco-régionale qui doit intégrer la dynamique des relations milieu/vecteur/parasite /maladie. Ces interactions sont complexes à Bandundu-ville et évoluent dans le sens de l'augmentation du paludisme. L’*Anopheles gambiae sl* s'est adapté dans cette écologie de Bandundu-ville [8, 40, 44].

## Conclusion

La diversité et la mosaïque des biotopes, la présence pérenne de l’*Anopheles gambiae sl* et les paramètres entomologiques de transmission du paludisme placent la ville de Bandundu dans une zone endémique stable. La participation de membres de la Communauté dans des programmes de gestion serait salutaire, une fois qu'ils comprennent le rôle qu'ils jouent dans la transmission du paludisme. Cette étude sur les paramètres entomologiques et écologiques dans la zone de Bandundu-ville, en République Démocratique du Congo, met à disposition un ensemble de données sur l'histoire naturelle du paludisme dans les zones tropicales d'Afrique. Si cette zone devait être retenue comme site sentinelle de PNLP, cette étude devrait servir d'une part lors de l’élaboration du plan stratégique de lutte, d'autre part lors de l'analyse finale comme base de comparaison avant l'intervention.
